# Supplementary material for: RNA export through the nuclear pore complex is directional
Source: Nat Commun. 2022 Oct 6;13:5881. doi: 10.1038/s41467-022-33572-7 (PMC9537521; doi:10.1038/s41467-022-33572-7)
Supplement: Supplementary file 1 — Supplementary Information [file 41467_2022_33572_MOESM1_ESM.pdf]

RNA export through the nuclear pore complex is directional

Supplementary information

Supplemental Figures

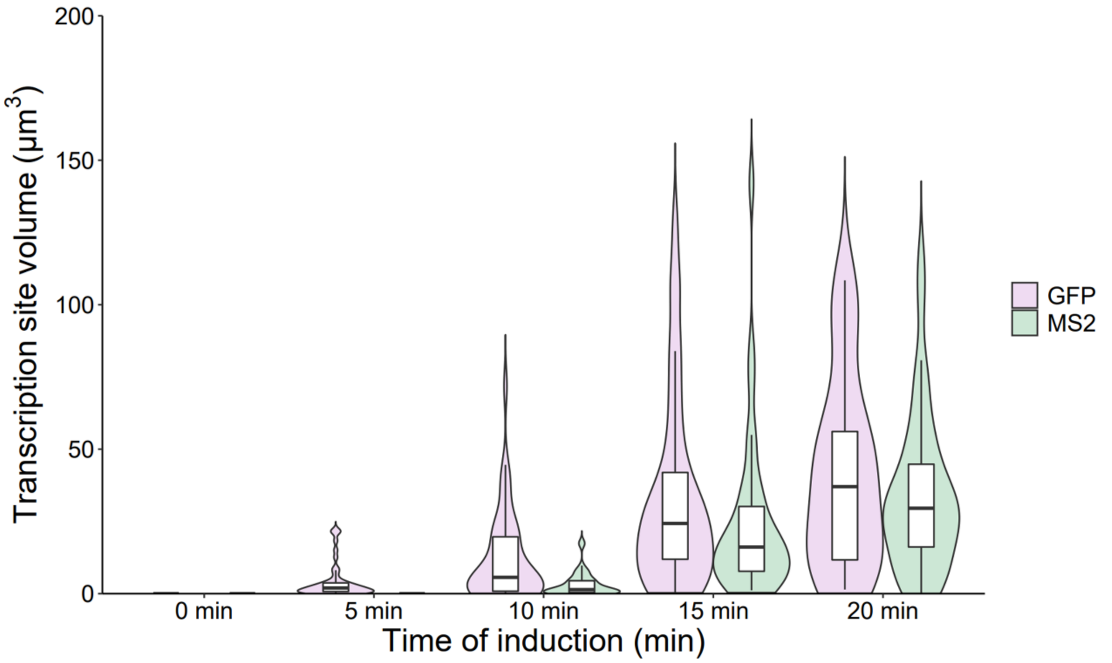

Supplementary Figure 1: Measurement of gene activation.

Violin plots depicting transcription site volume changes upon short activations using Ponasterone A to induce the GFP-Dys-MS2 gene. 5 min, GFP n=46, 10 min, GFP=44, MS2=44, 15 min, GFP=41, MS2=35, 20 min GFP=36, MS2=39 transcription sites (two-sided Mann-Whitney tests or one-sample Wilcoxon signed-rank tests against a value of 0 in cases of constant zero value, with Benjamini-Hochberg FDR correction, GFP: 0 vs. 5 min $p<0.0001$, 0 vs. 10 min $p<0.0001$, 0 vs. 15 min $p<0.0001$, 0 vs. 20 min $p<0.0001$, 5 vs. 10 min $p=0.0204$, 5 vs. 15 min $p<0.0001$, 5 vs. 20 min $p<0.0001$, 10 vs. 15 min $p=0.0002$, 10 vs. 20 min $p<0.0001$, 15 vs. 20 min $p=0.2152$. MS2: 0

vs. 10 min $p<0.0001$, 0 vs. 15 min $p<0.0001$, 0 vs. 20 min $p<0.0001$, 5 vs. 10 min $p<0.0001$, 5 vs. 15 min $p<0.0001$, 5 vs. 20 min $p<0.0001$, 10 vs. 15 min $p<0.0001$, 10 vs. 20 min $p<0.0001$, 15 vs. 20 min $p=0.045$. GFP vs. MS2: 5 min $p<0.0001$, 10 min $p=0.0099$, 15 min $p=0.3336$, 20 min=0.4357). Boxplots show the transcription site volume (μm^3) (center line, median; box limits, upper and lower quartiles; whiskers, 1.5× interquartile range).

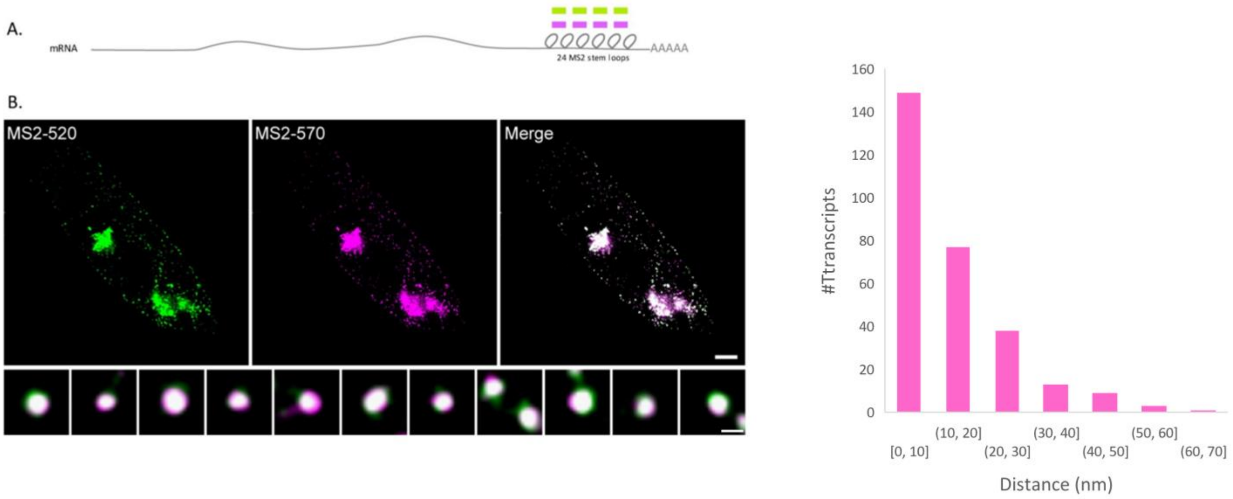

Supplementary Figure 2: Imaging controls and calibrations.

(**A**) Scheme of the GFP-Dys-MS2 construct, along with the FISH probes that hybridize with the MS2 region in the mRNA. (**B**) Transcripts tagged with two MS2 probe sets with the same sequences (magenta 570 nm and green 520 nm). Scale bars, 5 µm, 0.5 µm. A histogram depicting distance frequency in nm. n= 192 transcripts.

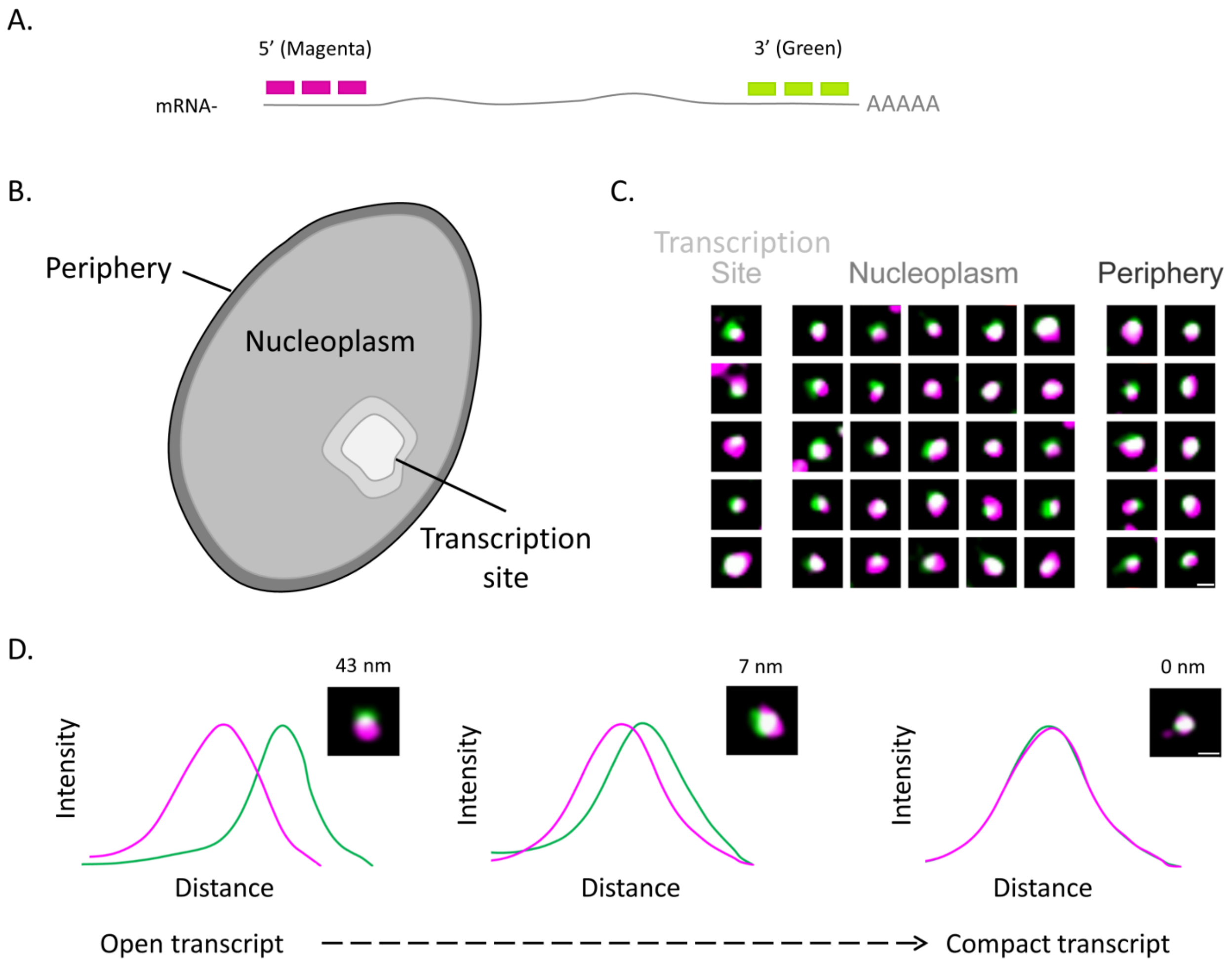

Supplementary Figure 3: mRNAs change their compaction state during travels throughout the nucleus.

(**A**) Scheme of the GFP-Dys-MS2 construct, along with the FISH probes that hybridize with different regions in the mRNA. (**B**) Scheme depicting the subnuclear areas analyzed. Transcription site- transcripts are up to 0.5 micron away from site; periphery- transcripts are up to 1 micron away from edge of Hoechst stain. (**C**) Transcripts imaged in different parts of the nucleus, 5'-end magenta, 3'-end green. (**D**) A scheme depicting the distance between fluorescence peaks as

measured by Leica LASX, representing 5’-3 ’distance in different transcripts. Adjustments of individual color channels were made.

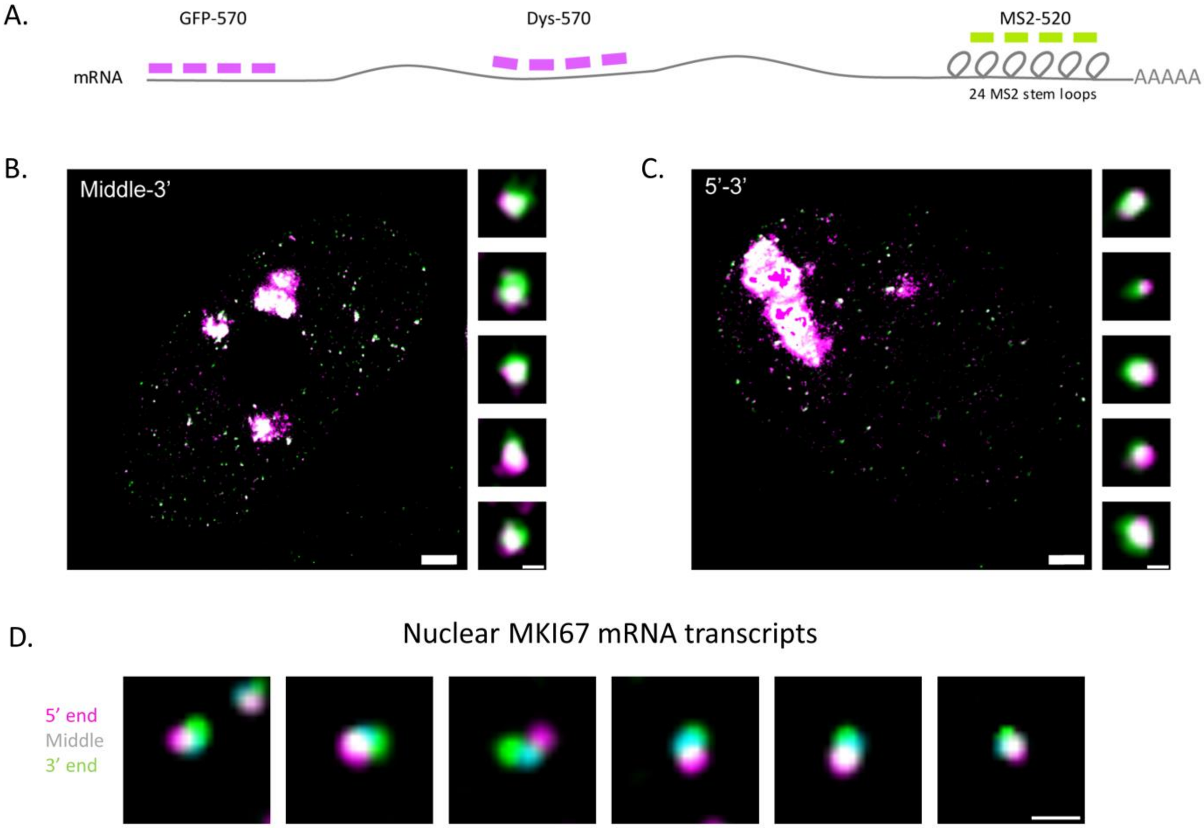

Supplementary Figure 4: Nucleoplasmic GFP-Dys-MS2 mRNA transcripts are rod-shaped.

(**A**) Scheme of the GFP-Dys-MS2 construct, along with the 5'-end, middle and 3'-end FISH probes used. Dystrophin probe binding sites are located ~5 kbp from the GFP sequence and ~6 kbp from the MS2 region, compared to the MS2 probes that are 11 kbp away from the GFP sequence. (**B**) Middle of the transcript was tagged using probes to the Dys region at 570 nm (magenta) and the 3'-end was tagged using probes to the MS2 region at 520 nm (green) probes. Large foci are the active transcription sites and the small dots are the mRNAs measured above. Right – enlarged examples of single mRNAs. Scale bars, 8 µm, 0.5 µm. (**C**) 5'-end (start) of the transcript was tagged using probes to the GFP region at 570 nm (magenta) and the 3'-end was tagged using MS2 probes

at 520 nm (green) probes. Large foci are the active transcription sites and the small dots are the mRNAs measured above. Right – enlarged examples of single mRNAs. Scale bars, 8 µm, 0.5 µm. **(D)** Triple tagged single MKI67 mRNA transcripts in the U2OS nucleus (5'- magenta, middle- cyan, 3'- green). Scale bar, 0.5 µm. Adjustments of individual color channels were made.

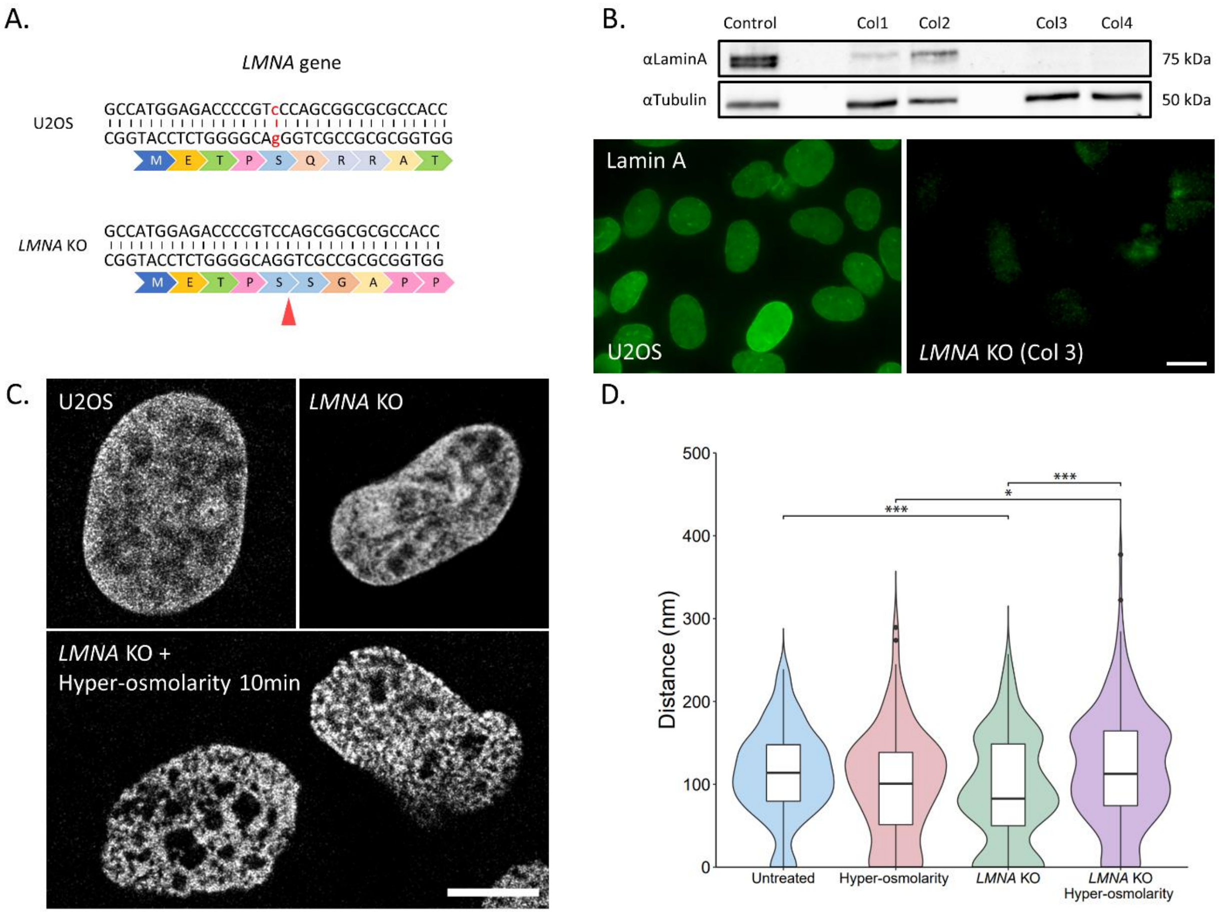

Supplementary Figure 5: The state of the genome organization affects nuclear mRNA compaction.

(**A**) sgRNA was directed to amino acid 5 of the *LMNA* gene to cause a frame shift mutation. (**B**) Top: Western blot to lamin A protein in U2OS cells compared knockout clones. Tubulin was used as a control. Bottom: Immunofluorescence for lamin A (green) in (left) U2OS compared to (right) *LMNA* knockout (KO) colony 3. Scale bar, 10 µm. (**C**) DNA (gray) in U2OS, *LMNA* KO and *LMNA* KO + hyper-osmolar treatment. Scale bar, 10 µm. (**D**) Violin plots comparing 5'-middle distances of

nuclear MKI67 transcripts under various conditions. U2OS n=171, *LMNA* KO (Col 3) n=246, *LMNA* KO + hyper-osmolar n=206 transcripts. (Kruskal-Wallis, $\chi^2(3)=23.732$, $p<0.0001$. Pairwise comparisons- two-sided Mann-Whitney tests with Benjamini-Hochberg FDR correction, Untreated vs. Hyper-osmolarity $p=0.093$, Untreated vs. *LMNA* KO $p=0.0008$, Untreated vs. *LMNA* KO Hyper-osmolarity $p=0.421$, Hyper-osmolarity vs. *LMNA* KO $p=0.3624$, Hyper-osmolarity vs. *LMNA* KO Hyper-osmolarity $p=0.02$, *LMNA* KO vs. *LMNA* KO Hyper-osmolarity $p=0.0001$). *$p<0.05$, ***$p<0.001$. Boxplots show the distance (nm) (center line, median; box limits, upper and lower quartiles; whiskers, 1.5× interquartile range; dots, outliers).

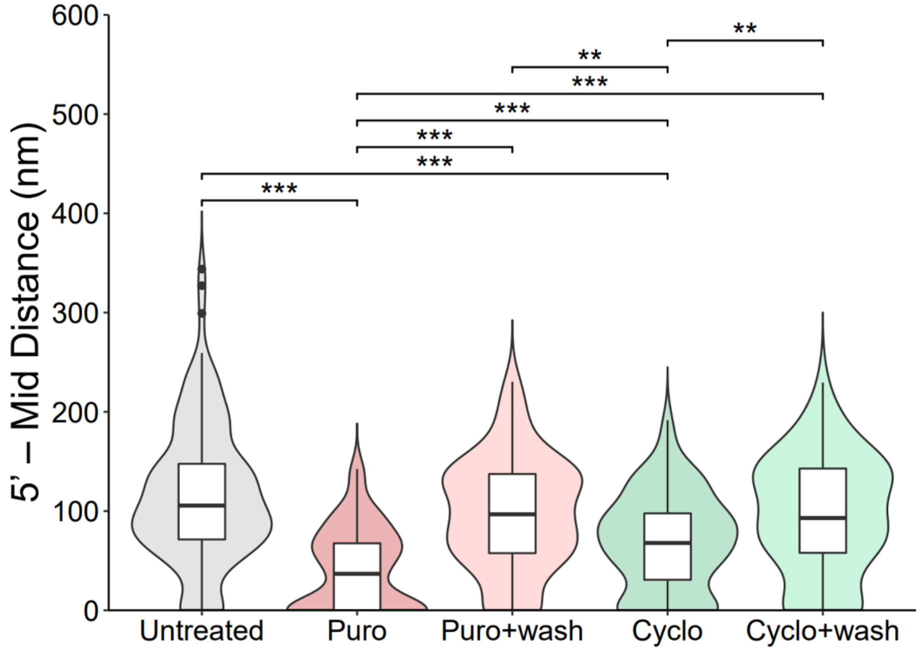

Supplementary Figure 6: Cytoplasmic MKI67 compaction changes upon translation inhibition, in a reversible manner.

Violin plots comparing MKI67 mRNA 5′-mid distance distribution for untreated, Puromycin, Puromycin + wash, Cycloheximide and Cycloheximide + wash-treated U2OS cells. No treatment n=128 mRNAs; Puromycin n=87 mRNAs; Puromycin + wash n=93 mRNAs; Cycloheximide n=94 mRNAs; Cycloheximide + wash n=64 mRNAs. (Kruskal-Wallis, $\chi^2(4)=84.414$, $p<0.0001$. Pairwise comparisons- two-sided Mann-Whitney tests with Benjamini-Hochberg FDR correction, Untreated vs. Puro $p<0.0001$, Untreated vs. Puro+Wash $p=0.0967$, Untreated vs. Cyclo $p<0.0001$, Untreated vs. Cyclo+Wash $p=0.13655$, Puro vs. Puro+Wash $p<0.0001$, Puro vs. Cyclo $p=0.0002$, Puro vs. Cyclo+Wash $p<0.0001$, Puro+Wash vs. Cyclo $p=0.001$, Puro+Wash vs. Cyclo+Wash $p=0.9972$, Cyclo vs. Cyclo+Wash $p=0.0067$).**$p<0.01$, ***$p<0.001$. Boxplots show the distance

(nm) (center line, median; box limits, upper and lower quartiles; whiskers, 1.5× interquartile range; dots, outliers).

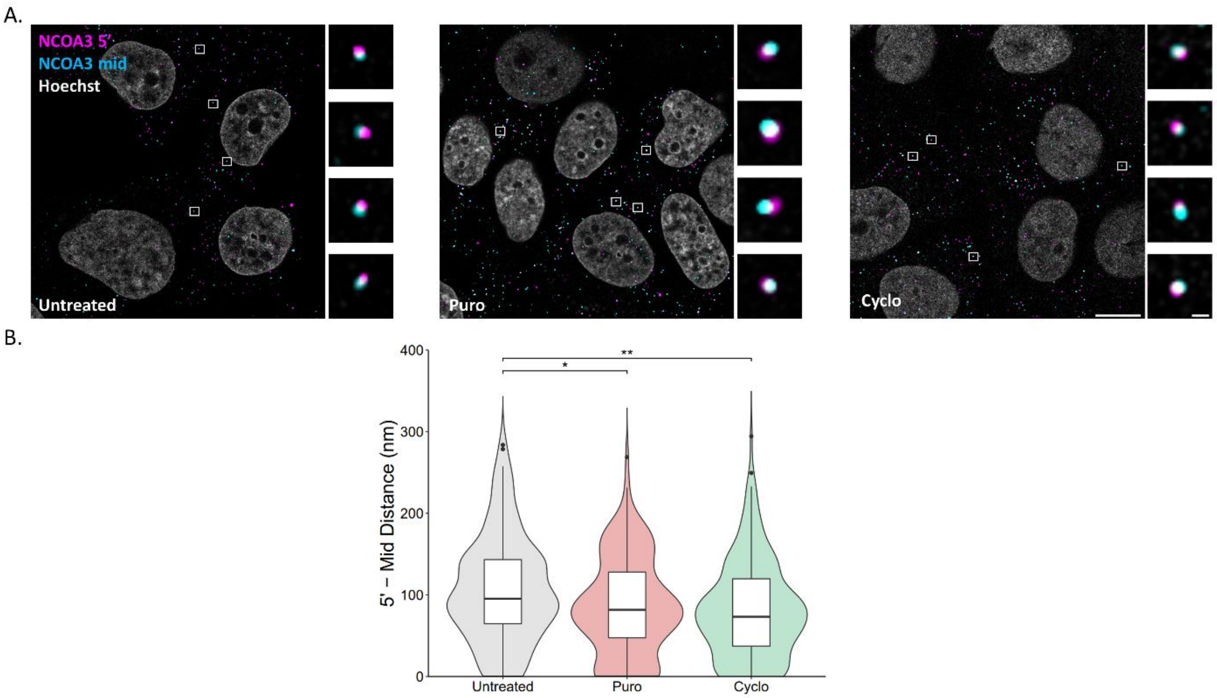

Supplementary Figure 7: Cytoplasmic NCOA3 compaction changes upon translation inhibition.

(**A**) Double tagging of NCOA3 mRNA transcripts in untreated, Puromycin and Cycloheximide-treated U2OS cells. Middle of the transcript was tagged using probes at 670 nm (cyan) and the 5ʾ-end was tagged using probes at 570 nm (magenta). Right – enlarged examples of single mRNAs. Scale bars, 8 µm, 0.5 µm. (**B**) Violin plots comparing NCOA3 mRNA 5′-mid distance distribution for untreated, Puromycin and Cycloheximide-treated U2OS cells. No treatment n=135 mRNAs; Puromycin n=139 mRNAs; Cycloheximide n=199 mRNAs. (Kruskal-Wallis, $\chi^2(2)=12.825$, $p=0.00164$. Pairwise comparisons- two-sided Mann-Whitney tests with Benjamini-Hochberg FDR correction, Untreated vs. Puro $p=0.0157$, Untreated vs. Cyclo $p= 0.0014$, Cyclo vs. Puro $p= 0.4997$). *$p<0.05$. **$p<0.01$. Boxplots show the distance (nm) (center line, median; box limits, upper and lower quartiles; whiskers, 1.5× interquartile range; dots, outliers).

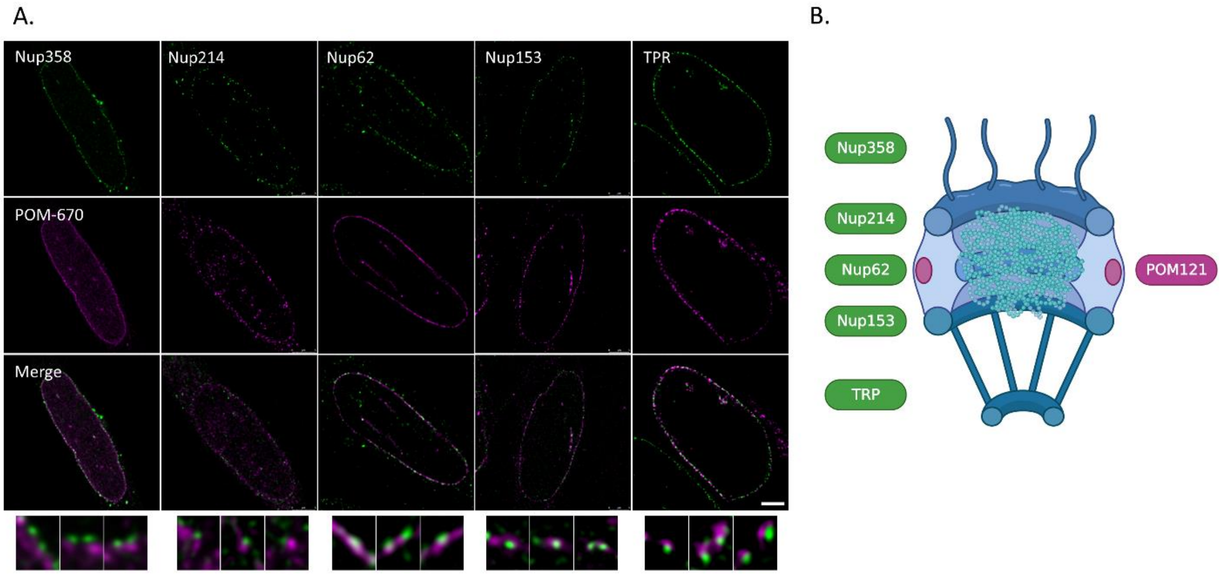

Supplementary Figure 8: Position of POM121 in the NPC.

(**A**) Confocal images showing POM121-RFP670 signal (magenta) compared to the different Nups (green). Enlarged examples of POM121-RFP670 and Nups are shown below. Scale bar, 10 µm. (**B**) A scheme (created with BioRender.com) representing the location of POM121 in the nuclear pore complex relative to other Nups. Adjustments of individual color channels were made.

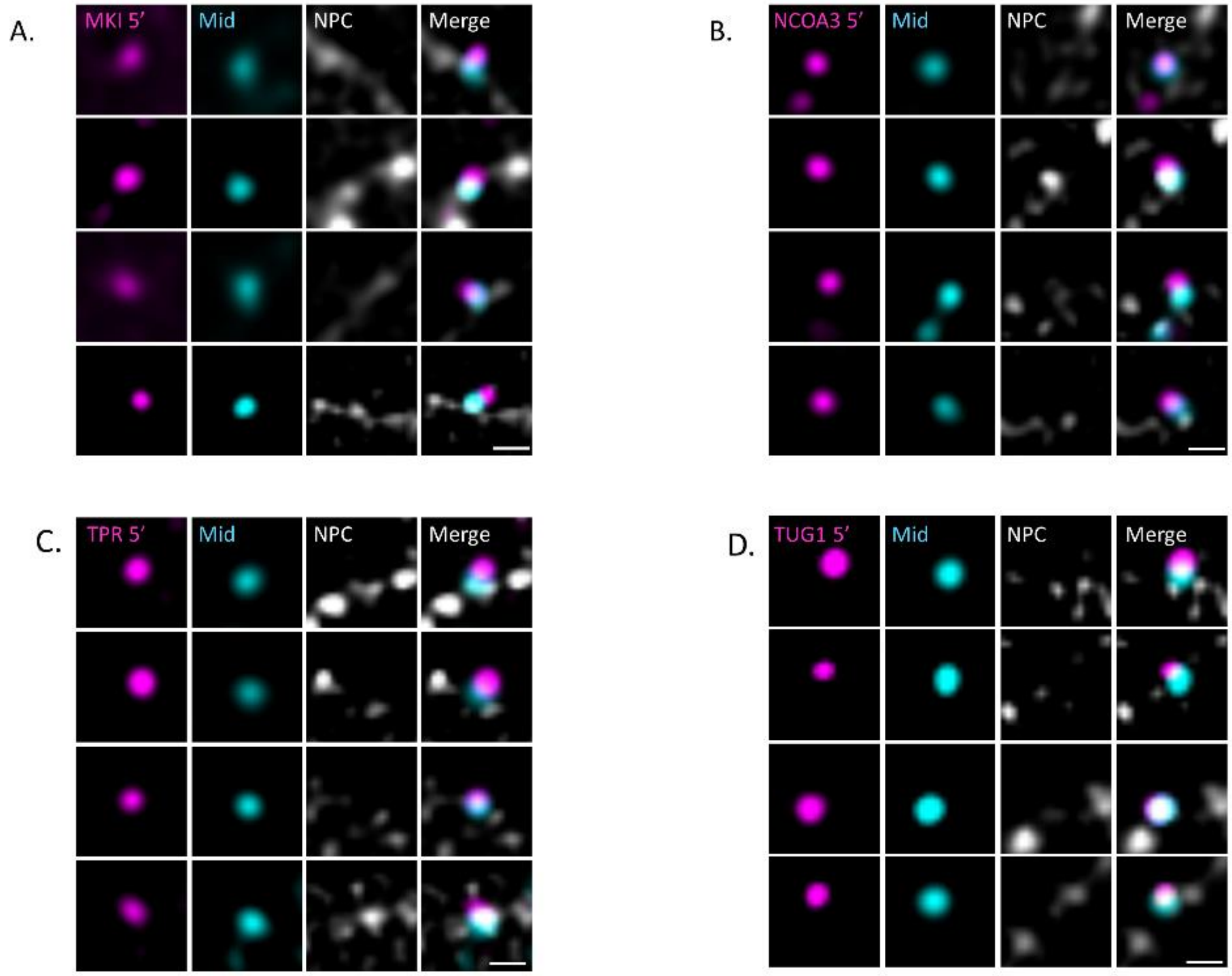

Supplementary Figure 9: mRNA and lncRNA export from the NPC in a 5’-first manner.

(**A**) MKI67, (**B**) NCOA3, (**C**) TPR and **(D**) TUG1 double-tagged transcripts in their 5' and middle regions (5’- magenta 570 nm, middle- cyan 670 nm) together with POM121-Cer labeling (gray). Scale bars, 0.5 µm.

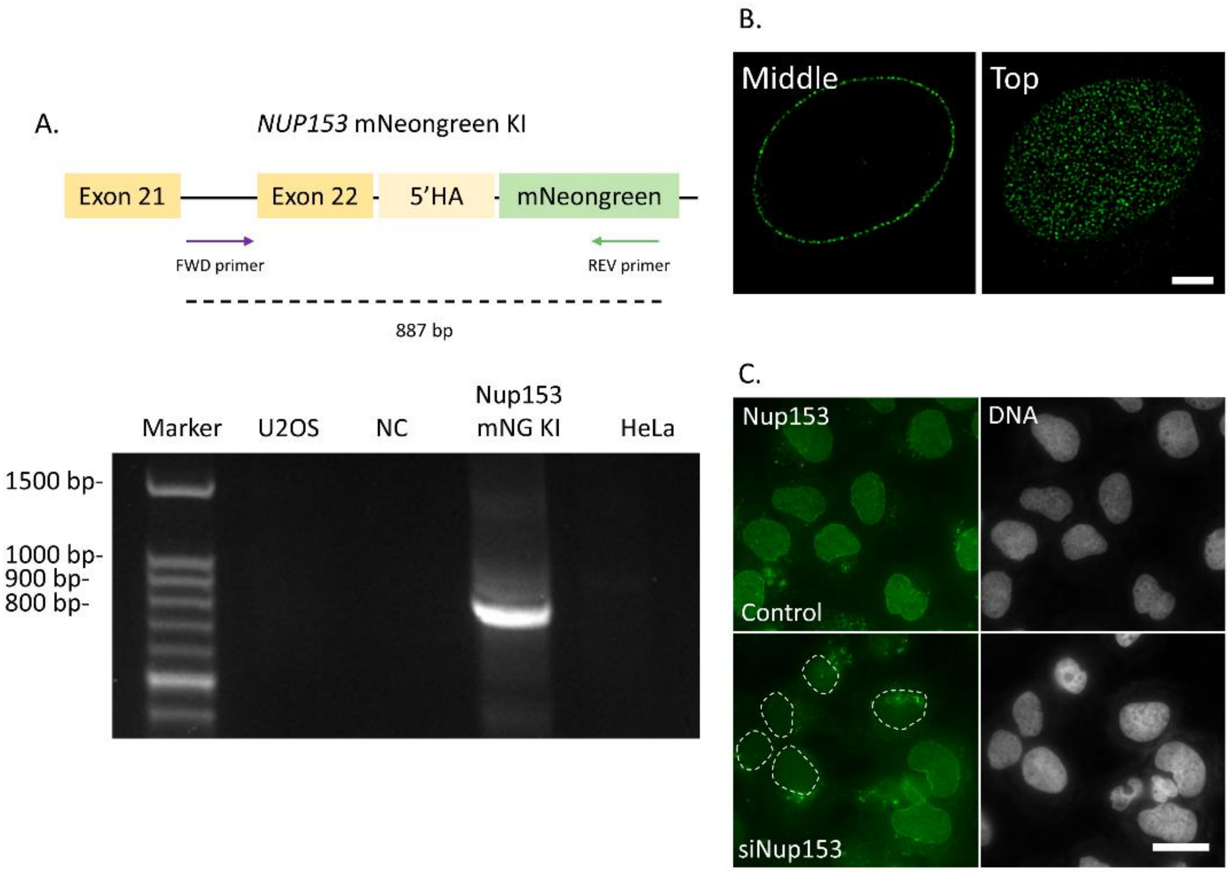

Supplementary Figure 10: mNeongreen knockin to *Nup153*.

(**A**) Scheme of primers used to characterize the genomic locus of *Nup153*. Genomic PCR results for the knock-in of a cassette containing mNeongreen into the 3'-end of the *Nup153* gene in U2OS cells. DNA from knockin (KI) cells was compared to control U2OS and HeLa cells. Primers used are described in methods; expected size 887 bp. (HA- homologous arm, NC- negative control, mNG- mNeongreen). (**B**) Middle and top planes of a Nup153-mNeongreen (green) expressing cell. Scale bar, 5 µm. (**C**) Nup153-mNeongreen stable cell line before (top) and after (bottom) siRNA knockdown of Nup153. Nuclei with reduced mNG fluorescence due to siNup153 are marked. Hoecsht DNA stain is in gray. Scale bar, 25 µm.

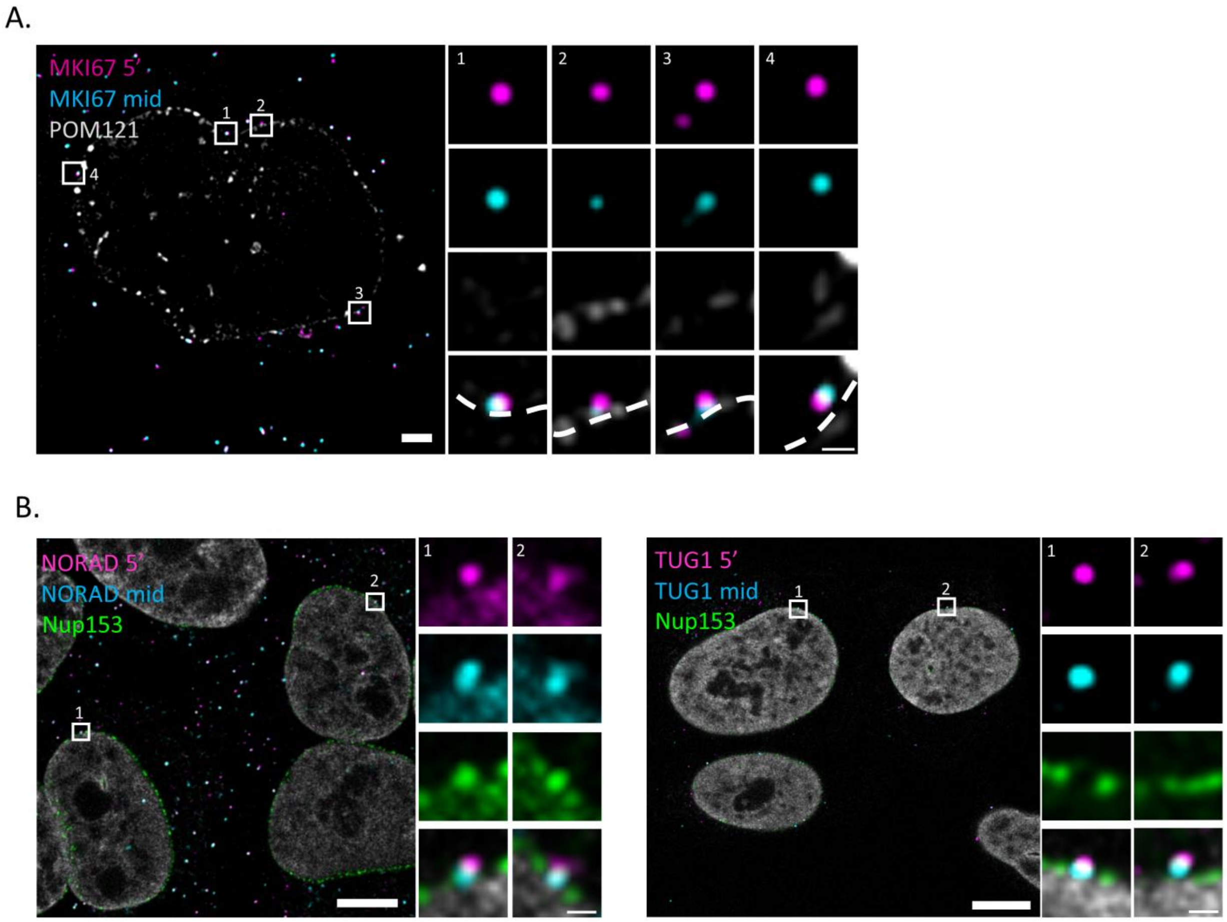

Supplementary Figure 11: Endogenous transcripts detected at the NPC.

(**A**) Double-tagged MKI67 transcripts (5'- magenta, middle- cyan) in a POM121-Cer expressing cell. Enlarged images depict 4 transcripts in or around the NPC. Dotted line shows the nuclear borders. Scale bar, 5 µm, 0.5 µm. (**B**) Left- Double-tagged NORAD transcripts (5'- magenta, middle- cyan) in a Nup153-mNeonGreen (green) expressing cell. Enlarged images depict 2 transcripts at the NPC. Right- Double-tagged TUG1 lncRNAs (5'- magenta, middle- cyan) in a Nup153-mNeonGreen (green) expressing cell. Enlarged images depict 2 transcripts at the NPC. Scale bars, 8 µm, 0.5 µm. Adjustments of individual color channels were made.

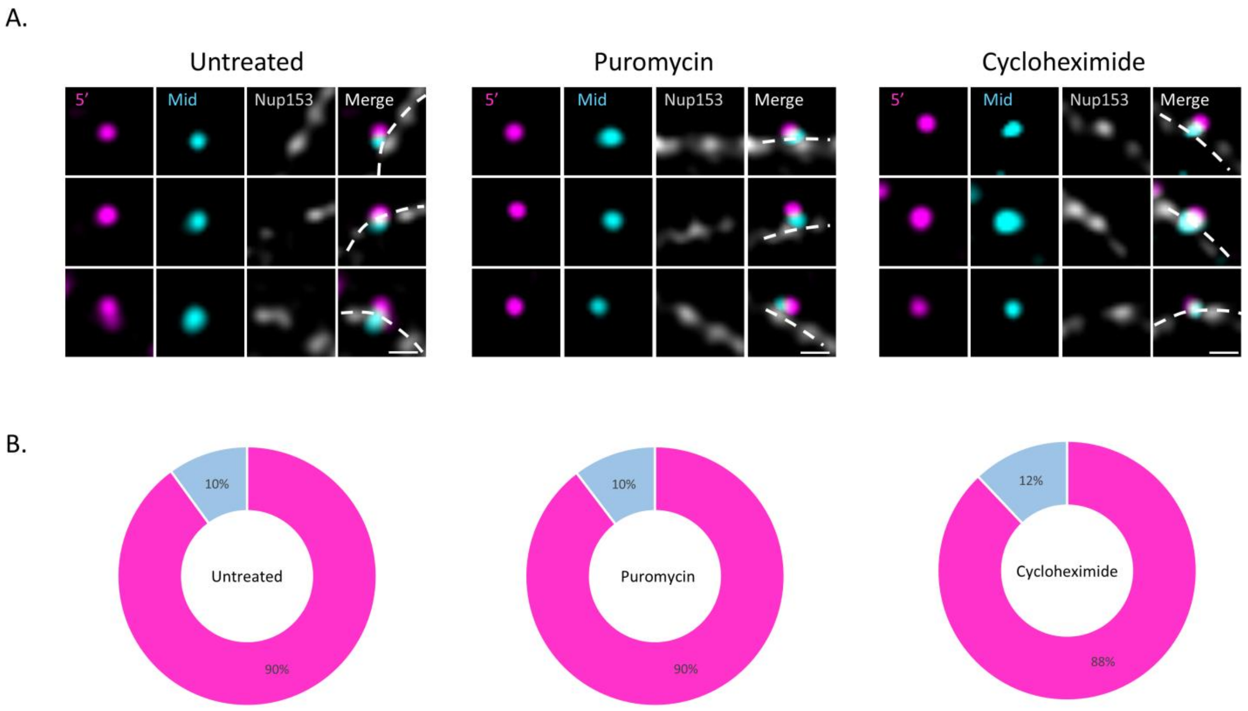

Supplementary Figure 12: mRNA export directionality is not affected by the state of translation.

(**A**) Double-tagged MKI67 transcripts (5'- magenta 570 nm, middle- cyan 670 nm) in a Nup153-mNeonGreen (green) expressing cell, under control or translation inhibition conditions Enlarged images depict transcripts at the NPC. Scale bar, 0.5 µm. (**B**) Charts describing the percentage of transcripts exiting the pore in a 5'-manner (magenta) or middle-first (cyan) in cells expressing Nup153-mNeonGreen. No treatment n=67 mRNAs; Puromycin n=58 mRNAs; Cycloheximide n=65 mRNAs. Adjustments of individual color channels were made.

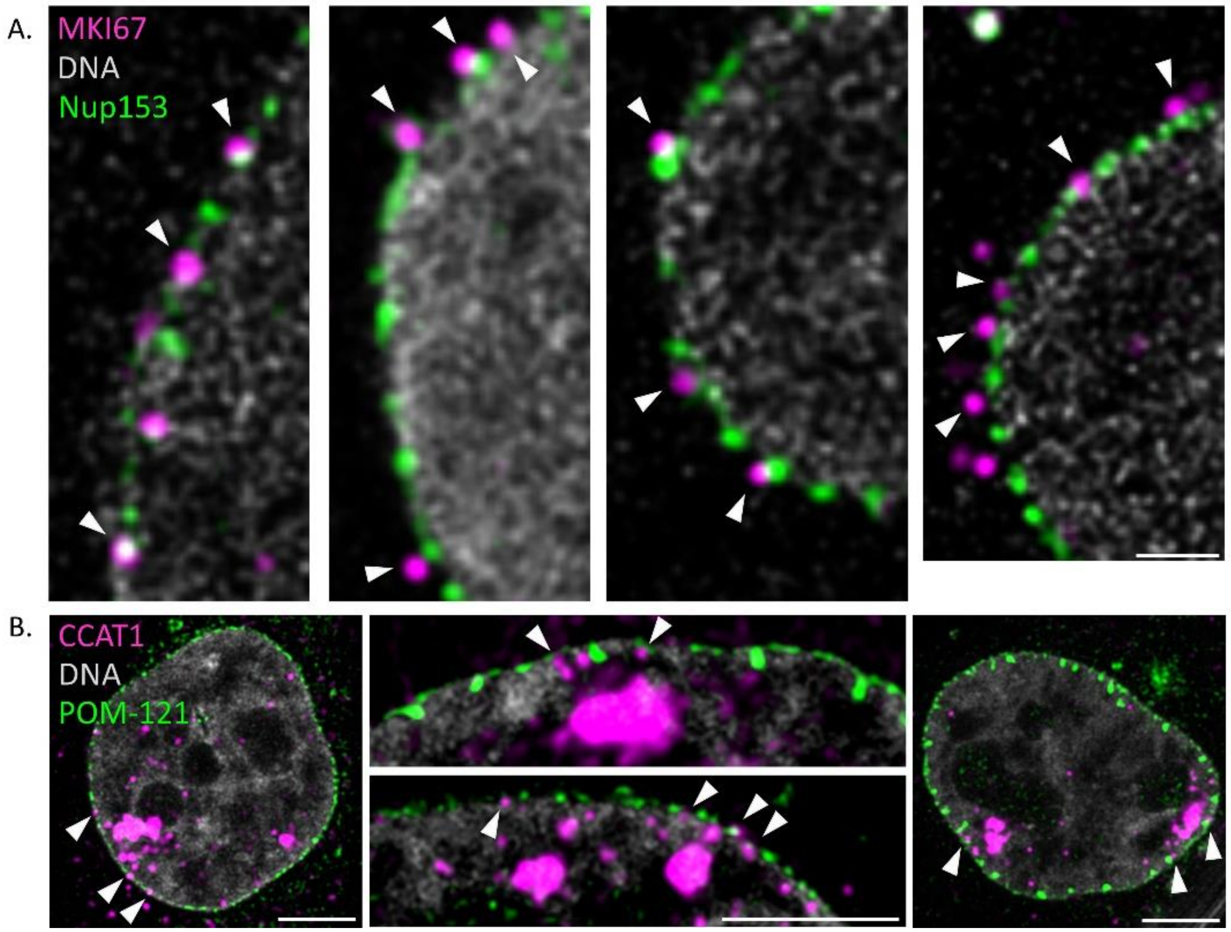

Supplementary Figure 13: Exported MKI67 and CCAT1 can be associated with adjacent NPCs.

(**A**) MKI67 mRNA transcripts (magenta, arrowheads) in the nuclei (Hoechst, gray) of U2OS Nup153-mNeongreen expressing cells (Green). Images depict several transcripts at adjacent NPCs. Scale bar, 5 µm. Adjustments of individual color channels were made. (**B**) CCAT1 lncRNAs (magenta, arrowheads, large spots are transcription sites) in the nuclei (Hoechst, gray) of POM121-Cer HeLa expressing cells (green). Images depict several transcripts at adjacent NPCs. Scale bar, 5 µm. Adjustments of individual color channels were made.
