# Supplementary material for: RNA export through the nuclear pore complex is directional
Source: Nat Commun. 2022 Oct 6;13:5881. doi: 10.1038/s41467-022-33572-7 (PMC9537521; doi:10.1038/s41467-022-33572-7)
Supplement: Supplementary file 3 — Description of Additional Supplementary Files [file 41467_2022_33572_MOESM3_ESM.pdf]

Description of Additional Supplementary Files

File Name: Supplementary Data 1
Description: List of probe sequences used for the detection of RNAs by RNA FISH. Each tab lists the probe set for a specific transcript and the region detected: 5'-end (5); middle (mid); 3'-end (3). If FLAP ends were added to the probes then they are noted as X or Y or Z.
