# Supplementary material for: RNA export through the nuclear pore complex is directional
Source: Nat Commun. 2022 Oct 6;13:5881. doi: 10.1038/s41467-022-33572-7 (PMC9537521; doi:10.1038/s41467-022-33572-7)
Supplement: Supplementary file 5 — Reporting Summary [file 41467_2022_33572_MOESM5_ESM.pdf]

nature portfolio

Corresponding author(s): Yaron Shav-Tal

Last updated by author(s): Aug 31, 2022

Reporting Summary

Nature Portfolio wishes to improve the reproducibility of the work that we publish. This form provides structure for consistency and transparency in reporting. For further information on Nature Portfolio policies, see our Editorial Policies and the Editorial Policy Checklist.

Statistics

For all statistical analyses, confirm that the following items are present in the figure legend, table legend, main text, or Methods section.

n/a	Confirmed	
☐	☒	The exact sample size (*n*) for each experimental group/condition, given as a discrete number and unit of measurement
☐	☒	A statement on whether measurements were taken from distinct samples or whether the same sample was measured repeatedly
☐	☒	The statistical test(s) used AND whether they are one- or two-sided<br>*Only common tests should be described solely by name; describe more complex techniques in the Methods section.*
☐	☒	A description of all covariates tested
☐	☒	A description of any assumptions or corrections, such as tests of normality and adjustment for multiple comparisons
☐	☒	A full description of the statistical parameters including central tendency (e.g. means) or other basic estimates (e.g. regression coefficient) AND variation (e.g. standard deviation) or associated estimates of uncertainty (e.g. confidence intervals)
☐	☒	For null hypothesis testing, the test statistic (e.g. *F*, *t*, *r*) with confidence intervals, effect sizes, degrees of freedom and *P* value noted<br>*Give P values as exact values whenever suitable.*
☒	☐	For Bayesian analysis, information on the choice of priors and Markov chain Monte Carlo settings
☒	☐	For hierarchical and complex designs, identification of the appropriate level for tests and full reporting of outcomes
☒	☐	Estimates of effect sizes (e.g. Cohen's *d*, Pearson's *r*), indicating how they were calculated

Our web collection on statistics for biologists contains articles on many of the points above.

Software and code

Policy information about availability of computer code

Data collection	Olympus CellR, Leica SP8
Data analysis	Huygens Essential/professional (STED)<br>ImageJ Java.1.52a<br>LASX<br>IMARIS 9.7.2<br>StudioR<br>Excel 2019<br>Clone Manager 11

For manuscripts utilizing custom algorithms or software that are central to the research but not yet described in published literature, software must be made available to editors and reviewers. We strongly encourage code deposition in a community repository (e.g. GitHub). See the Nature Portfolio guidelines for submitting code & software for further information.

Data

Policy information about availability of data

All manuscripts must include a data availability statement. This statement should provide the following information, where applicable:

- Accession codes, unique identifiers, or web links for publicly available datasets
- A description of any restrictions on data availability
- For clinical datasets or third party data, please ensure that the statement adheres to our policy

HGCr38 dataset was used for CRISPR and probe design. The datasets generated during and/or analysed during the current study are available from the corresponding author on reasonable request

Field-specific reporting

Please select the one below that is the best fit for your research. If you are not sure, read the appropriate sections before making your selection.

☒ Life sciences ☐ Behavioural & social sciences ☐ Ecological, evolutionary & environmental sciences

For a reference copy of the document with all sections, see nature.com/documents/nr-reporting-summary-flat.pdf

Life sciences study design

All studies must disclose on these points even when the disclosure is negative.

Sample size	No statistical methods were used to pre-determine sample size. Sample size of all experiments was based on the extensive experience in the lab in quantifying single RNAs and in similar published research (doi: 10.1016/j.molcel.2018.10.010), to ensure adequate statistical power. In addition, the sample sizes for each experiment have been detailed in the figure legends and methods and confirmed statistically by appropriate tests.
Data exclusions	Transcripts which were not triple tagged were excluded from triple-tag measurements. Transcripts which exhibited colocalized 5' and mid signals were excluded from directionality analysis.
Replication	The experimental findings reported in this manuscript were reliably reproducible (at least 3 repeats) and attempts at replication were successful.
Randomization	Not relevant to this type of study- each treatment was done for different times or in different mediums, and so the relevant experiments were analyzed blindly rather than randomized.
Blinding	In treatment experiments (ATP depletion, translation inhibition, hyper osmolation, LMNA KO) analysis was performed along with control and blindly. During data analysis of these experiments, the investigators were blinded to which treatment they were analyzing.

Reporting for specific materials, systems and methods

We require information from authors about some types of materials, experimental systems and methods used in many studies. Here, indicate whether each material, system or method listed is relevant to your study. If you are not sure if a list item applies to your research, read the appropriate section before selecting a response.

Materials & experimental systems

n/a	Involved in the study	
☐	☒	Antibodies
☐	☒	Eukaryotic cell lines
☒	☐	Palaeontology and archaeology
☒	☐	Animals and other organisms
☒	☐	Human research participants
☒	☐	Clinical data
☒	☐	Dual use research of concern

Methods

n/a	Involved in the study	
☒	☐	ChIP-seq
☒	☐	Flow cytometry
☒	☐	MRI-based neuroimaging

Antibodies

Antibodies used	All antibodies used were supplied by Abcam, unless specified otherwise. rabbit anti-Nup358 at 1:200 (ab64276); rabbit anti-Nup214 at 1:200 (ab70497); mouse anti-Nup62 at 1:300 (ab96134); rabbit anti-Nup153 at 1:300 (ab171074); rabbit anti-TPR at 1:100 (ab170940); rabbit anti-LaminA at 1:200 (ab26300); rabbit anti-Tubulin at 1:10000 (ab4074).<br>Secondary antibodies (all 1:1000): tanti-mouse Cy3 (ab97035), anti-rabbit Cy3 (ab6939), Alexa Fluor 647 goat anti-mouse (A21235; Molecular Probes), and Alexa Fluor 594 goat anti-rabbit (A11072; Molecular Probes).

Validation	The specificity of primary antibodies was validated by the manufacturer.

Eukaryotic cell lines

Policy information about cell lines

Cell line source(s)	U2OS, HeLa, MCF7 cell lines were obtained from the ATCC, U2OS Tet-On cell line was obtained from Clontech.
Authentication	Cell lines were authenticated by manufacturer. U2OS and HeLa cells were recently authenticated by STR profiling.
Mycoplasma contamination	All cell lines tested negative for mycoplasma contamination
Commonly misidentified lines (See ICLAC register)	No commonly misidentified cell line were used
